# Structural Concepts, Definition, Classification, and Macronutrient and Food Composition of Carbohydrate-Restricted Diets for Individuals with Type 2 Diabetes Mellitus: A Scoping Review

**DOI:** 10.3390/nu17061061

**Published:** 2025-03-18

**Authors:** Fharlley Lohann Medeiros, Ana Carolina Fernandes, Mariana V. S. Kraemer, Marina Padovan, Greyce Luci Bernardo, Paula Lazzarin Uggioni, Alex Rafacho, Rossana P. C. Proença

**Affiliations:** 1 Graduate Program in Nutrition and Nutrition in Foodservice Research Centre, Federal University of Santa Catarina (UFSC), Florianópolis 88040-370, SC, Brazil; nutrilohann@gmail.com (F.L.M.); ana.fernandes@ufsc.br (A.C.F.); marianavskraemer@gmail.com (M.V.S.K.); padovanmarina.luiz@gmail.com (M.P.); greyce.bernardo@ufsc.br (G.L.B.); paula.uggioni@ufsc.br (P.L.U.); 2 Laboratory of Investigation in Chronic Diseases, Center of Biological Sciences, Federal University of Santa Catarina (UFSC), Florianópolis 88037-000, SC, Brazil; alex.rafacho@ufsc.br

**Keywords:** dietary guidelines, dietary intervention, glycemic control, ketogenic diet, low-carbohydrate diet

## Abstract

**Objective:** This study aimed to review the structural concepts, definition, classification, and macronutrient and food composition of carbohydrate-restricted diets (CRDs) for individuals with type 2 diabetes mellitus (T2DM). **Methods:** A scoping review was conducted following Joanna Briggs Institute guidelines. Searches were performed in Scopus, PubMed, Web of Science, and Embase, including texts published in Portuguese, English, and Spanish. Official documents from governments, regulatory agencies, and international diabetes organizations were also consulted. **Results:** In total, 79 articles and 17 official documents were analyzed. The following structural concept was identified: restricted carbohydrate intake decreases the need for endogenous and exogenous insulin, contributing to the maintenance of glycemic control, and justifies its consideration among the nutritional therapy options for individuals with T2DM. CRDs varied in definition, classification, and macronutrient composition. Studies failed to provide detailed information on the food composition of diets, precluding an in-depth understanding of metabolic effects. The existence of several approaches with varying recommendations makes it difficult to generalize the results. International CRD guidelines for T2DM adopt divergent definitions, compromising interpretation, recommendation, and even adherence. **Conclusions:** Although the concept of CRDs justifies their adoption within the nutritional therapy choices for T2DM, the multiple denominations can hinder understanding and comparison between studies. The lack of information on food composition and carbohydrate types compromises the assessment of the effects and adherence to CRD-based nutritional interventions. We emphasize the need for methodologically consistent studies that evaluate CRDs based on fresh and minimally processed foods with a low glycemic index to support official diabetes guidelines and organizations.

## 1. Introduction

Diabetes mellitus comprises a heterogeneous group of metabolic disorders characterized by hyperglycemia, resulting from the deficient production and/or action of the hormone insulin [1]. Type 2 diabetes mellitus (T2DM) is the most prevalent form of the disease, accounting for more than 90% of diabetes cases worldwide [2]. Currently, there is no unified dietary standard for the management of T2DM.

Carbohydrate-restricted diets (CRDs), among other eating patterns, have been mentioned in consensus reports as one of the choices for individuals with T2DM by global experts and associations, such as the American Diabetes Association (ADA) and the European Association for the Study of Diabetes (EASD), highlighting, among other benefits, the reduction of metabolic demand for insulin, improvement in glycemic control, minimization of postprandial glycemic spikes, decrease in the occurrence of hypoglycemic events, and reduction in the frequency and use of hypoglycemic medications and insulin [3,4].

However, some challenges associated with these diets have been reported, such as difficulties in defining CRDs, low adoption, and poor long-term adherence [1,5,6,7,8]. Systematic review studies revealed a variety of definitions and a lack of consensus concerning the conceptualization of CRDs, underscoring that this inconsistency may explain the controversial results, as it makes it difficult to draw comparisons [9,10,11,12,13,14,15,16,17,18,19,20]. Furthermore, these challenges can hinder the development of recommendations in diabetes guidelines and other official documents.

Despite the relevance of the theme, no literature review studies were found that analyzed the aforementioned issues related to CRDs for T2DM. In view of this gap, this study aimed to review the structural concepts, definition, classification, and macronutrient and food composition of CRDs for individuals with T2DM, contributing to the creation of a scientific frame of reference.

## 2. Materials and Methods

### 2.1. Study Design

A scoping review was conducted following the recommendations of the Joanna Briggs Institute (JBI) [21]. The findings are reported based on the Preferred Reporting Items for Systematic Reviews and Meta-Analyses—Extension for Scoping Reviews (PRISMA-ScR) [22]. The study protocol was registered on the OSF platform (https://osf.io/) under doi.org/10.17605/OSF.IO/KYXJZ, accessed on 14 February 2025.

### 2.2. Eligibility Criteria

The research question was formulated based on the Population—Concept—Context (PCC) framework [21], as follows: Population, individuals with T2DM; Concept, carbohydrate-restricted diets—CRDs; and Context, structural concepts, definition, classification, and macronutrient and food composition of diets in carbohydrate-based recommendations for the treatment of T2DM. The following inclusion criteria were adopted: texts published in Portuguese, English, or Spanish; qualitative and quantitative empirical articles; and studies and official documents from governments, organizations, and regulatory agencies addressing the structural concepts, definition, and classification of macronutrients and foods of carbohydrate recommendations in the treatment of T2DM. Exclusion criteria were documents focused on individuals with prediabetes or types of diabetes mellitus other than T2DM, those including fasting or physical activity as intervention variables, and those not available in full text.

### 2.3. Literature Search

A systematic search was carried out in October 2022 in the Scopus, PubMed, Web of Science, and Embase databases. The search was updated in February 2024. Official documents from governments, regulatory agencies, and organizations were also consulted, including the ADA, the Brazilian Diabetes Society (SBD), Diabetes UK (DUK), Diabetes Canada, and the Royal Australian College of General Practitioners (RACGP). An additional search was carried out in Google Scholar. The reference lists of consulted documents were screened to identify other official texts from governments, organizations, and regulatory agencies.

Database searches were conducted using the following search strategy and terms: (Diabetes OR “Diabetes Mellitus” OR “Type 2 Diabetes Mellitus” OR t2d OR “non insulin-dependent diabetics”) AND ((“Carbohydrate-Restricted diet” OR “Low Carbohydrate diet” OR “High-protein low-carbohydrate diet” OR “Carbohydrate-restricted dietary pattern” OR “Carbohydrate restriction” OR Vlckd OR “Low carb diet” OR “High-protein low-carbohydrate diet” OR “Low-carbohydrate high-protein diet” OR “Carbohydrate-restricted high-protein diet” OR “Atkins diet” OR “South Beach diet” OR “Ketogenic diet”)).

### 2.4. Study Selection

Results retrieved from database searches were grouped using EndNote 20 software (Clarivate Analytics, Philadelphia, PA, USA). After duplicate exclusion, the titles, abstracts, and keywords of the remaining articles were screened for relevance. Then, the retained articles were read in full, and those that did not meet the inclusion criteria were excluded. One author (FLMRS) reviewed the title and abstracts of all articles entered in EndNote 20 (Clarivate Analytics, PA, USA). Subsequently, the same author screened the full texts and discussed the results with three other authors (MP, ACF, and RPC). Any divergence was discussed with the research team until consensus was reached.

### 2.5. Data Extraction and Analysis

The data were extracted from the selected studies and official documents into a Microsoft Word form using a spreadsheet previously developed by two authors (FLM and MP) and discussed with two other authors (ACF and RPCP). Information was gathered on authorship, year of publication, country of origin, objectives, methodology (study type, design, data collection method, and sample size), and dietary recommendations (structural concepts, definition, classification, carbohydrate content, and food composition of CRDs).

Data analysis was conducted using narrative synthesis. Information from different articles and official documents was synthesized into tables and figures, depending on the objective.

## 3. Results and Discussion

**Figure 1:**
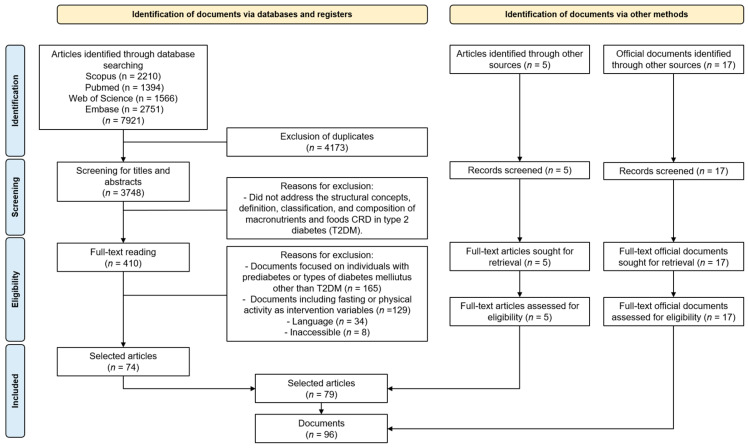
Flowchart detailing the steps in document selection for the scoping review on carbohydrate-restricted diets (CRDs) for individuals with type 2 diabetes mellitus (T2DM).

A total of 7921 articles were identified in the initial search. After duplicates were excluded, 3748 documents remained and were screened by title and abstract. In the subsequent screening step, 410 documents were read in full. The application of inclusion criteria led to the selection of 74 studies. The search for official documents and other sources retrieved 22 documents, which were included in the study. The document selection flowchart is depicted in Figure 1.

At the end of the assessment, 96 documents were selected for the scoping review, including 79 articles and 17 official documents (Table S1, Supplementary Material) addressing the structural concepts, definition, classification, and macronutrient and food composition of CRDs for individuals with T2DM.

### 3.1. Structural Concepts Informing Recommendations of CRDs for Individuals with T2DM

The recommendation of CRDs as a nutritional approach to the management of T2DM is based on the premise that restricted carbohydrate intake reduces the metabolic demand for insulin, contributing to the control of glycemic homeostasis [23]. This strategy consists of limiting simple and refined carbohydrates and prioritizing sources of complex carbohydrates, such as whole grains, fibrous non-starchy vegetables, and low-glycemic fruits [4,17]. Moreover, the reduced carbohydrate intake provided by CRDs contributes to minimizing postprandial glycemic spikes, decreasing the occurrence of hypoglycemic events, and potentially reducing the frequency and concentration of medications for glycemic control [23,24].

The human body’s responses to carbohydrate restriction include improved glycemic control and reduced hyperinsulinemia, both of which depend on carbohydrate intake. Accordingly, the lower the dietary intake of carbohydrates, the greater the improvement in these parameters. These physiological changes stem from reduced exposure to the anabolic action of insulin, which promotes a shift toward a catabolic state that leads to fat oxidation and may even induce ketogenesis, depending on the intensity of carbohydrate restriction [25,26].

Ketogenesis refers to the production of ketone bodies from fatty acids by the liver, a process activated under conditions of low carbohydrate availability, particularly when glycogen stores are depleted. This metabolic route forms the basis of the ketogenic diet, a CRD that seems to have an appetite-suppressant effect, thereby simultaneously contributing to reducing body weight and controlling glycemia [27]. Studies have shown that individuals with T2DM who adhered to the ketogenic diet obtained health benefits beyond weight loss and improved glycemic control, such as requiring reduced doses of medications [11,28,29].

CRDs have historically been used for the treatment of diabetes, and this practice is still supported by basic biochemistry [30]. Feinman [30] argued that such diets should be considered as the first-line treatment for individuals with glucose intolerance or insulin resistance. The author also emphasized that a major barrier to our current understanding of the importance of CRDs in the treatment of individuals with T2DM is the lack of consensual definitions.

### 3.2. Definition, Classification, and Macronutrient and Food Composition of CRDs

Recommendations for carbohydrate restriction in individuals with diabetes mellitus date back to the 18th century [31]. Since then, carbohydrate restriction has been advocated for individuals with T2DM, under different terminologies, classifications, and macronutrient compositions.

In 2004, the ADA stated that CRDs (referred to in the document as low-carbohydrate diets) were not recommended for the treatment of diabetes, nor was it advisable that individuals with diabetes restricted their total carbohydrate intake to less than 130 g/day. Although the optimal proportion of carbohydrates for supporting human health had not been defined, it was recommended to follow a diet containing between 45% and 65% of the total energy value from carbohydrates, according to guidelines published by the Food and Nutrition Board of the National Academy of Sciences [32].

The minimum carbohydrate intake of 130 g/day is partly due to the fact that glucose is the brain’s main energy substrate. However, the body is equipped with numerous metabolic processes to meet this energy demand under conditions of low carbohydrate intake, such as glycogenolysis, gluconeogenesis, and ketogenesis [33]. Despite the recognition of metabolic processes that support low carbohydrate levels, many diets recommend restricting carbohydrate intake to levels not lower than 130 g/day [34].

Halton et al. [35,36] proposed a method for defining CRDs, which they named the low-carbohydrate diet score. This scoring system was initially applied to measure the risk of coronary heart disease and T2DM in women. The low-carbohydrate diet score is calculated based on the energy percentage derived from fats, proteins, and carbohydrates. Fat and protein intakes contribute positively to the score, whereas carbohydrate intake has the opposite effect.

According to the definition proposed by the American Academy of Family Physicians in 2006, CRDs (referred to as low-carbohydrate diets in the document) are characterized by restricted caloric intake and a reduction in carbohydrate intake to levels ranging from 20 to 60 g/day, which usually represents less than 20% of total energy intake. In these types of diets, the consumption of proteins and fats is increased to partially compensate for the calories lost from carbohydrates [37].

CRDs with a carbohydrate intake of 20 g/day are classified as very-low-carbohydrate diets and are capable of inducing ketosis. CRDs containing higher levels of carbohydrates, between 40 and 60 g/day, are not considered prone to inducing ketosis and are classified as low-carbohydrate diets [38]. Whereas some CRDs are defined by specific carbohydrate intake levels, typically below 70 g/day, others are based on the proportion of total energy intake, as shown in Table 1.

It should be noted that as the total energy value of the diet decreases, the proportion of energy derived from carbohydrates increases. For example, a diet containing 200 g of carbohydrates can be considered moderately low in carbohydrates for a 2000 kcal energy intake, moderate in carbohydrates for a 1500 kcal energy intake, and high in carbohydrates for a 1200 kcal energy intake [34]. When the carbohydrate intake is reduced, there is an increase in the fat and protein contents of the diet, resulting in a low-carbohydrate hyperproteic diet or non-ketogenic low-carbohydrate high-fat diet.

Fat and protein levels may vary in non-ketogenic low-carbohydrate diets, but are often higher than the reference daily intakes recommended for the general population. A very-low-carbohydrate diet typically has a carbohydrate content ranging from 20 to 50 g/day and is high in fat and/or protein. The Atkins diet, for example, contains 35% protein and 50% fat, deriving only 10% of the total energy value from carbohydrates [39]. Additionally, it is commonly considered a ketogenic diet, and 60% or more of the total energy value may be sourced from fat. It should be noted that the carbohydrate threshold for the induction of ketogenesis varies among individuals but is estimated at 50 g/day or 10% of a 2000 kcal diet [23,40,41,42]. On the other hand, a very-low-carbohydrate hyperproteic diet is composed of 25% to 35% fat and 55% to 65% protein [39].

In 2007, the ADA defined CRDs (referred to as low-carbohydrate diets) as diets that restricted total carbohydrates to less than 130 g/day or 26% of a 2000 kcal diet. In 2015, Feinman and colleagues indicated that such carbohydrate recommendations would be far from a restriction, given that “before the increase in obesity prevalence in the USA, the average carbohydrate intake was 43% of total energy and, at present, a carbohydrate intake of 26% to 45% of total energy is recommended for moderate carbohydrate diets” [24].

Although there is no universal consensus on what constitutes a CRD, most studies adopt the definitions and classifications proposed by Feinman et al. [24]. The authors proposed a terminology for diets with different carbohydrate contents, which are categorized into very-low-, low-, moderate-, or high-carbohydrate diets (Table 1). The proposed classification system is based on experimental studies [23,30,43,44], daily carbohydrate intakes, and the corresponding percentages for a 2000 kcal diet.

**Figure 2:**
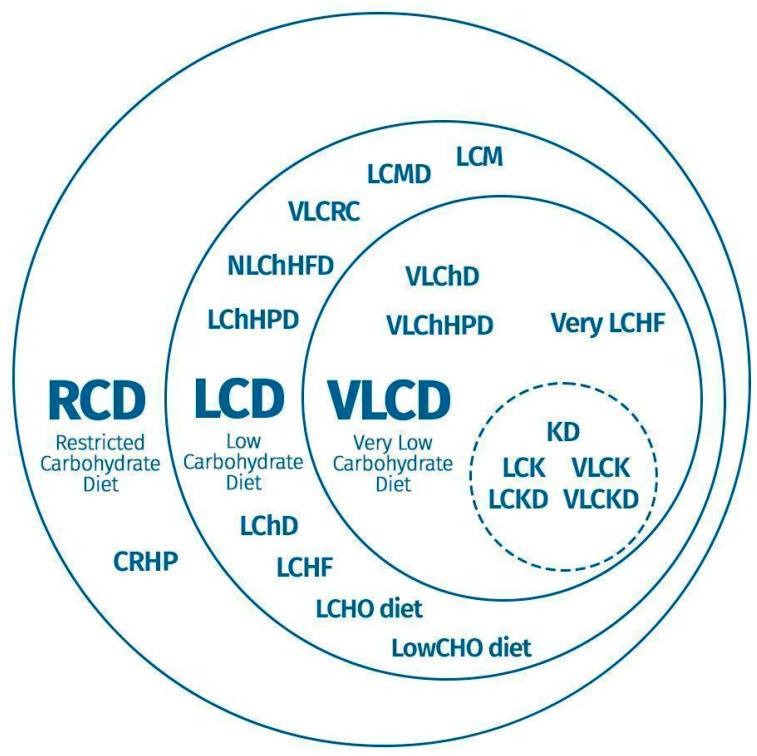
Variability in acronyms designating carbohydrate-restricted diets. Legend: RCD, restricted carbohydrate diet; CRHP, carbohydrate-restricted high-protein diet; LCD, low-carbohydrate diet; LChHPD, low-carbohydrate hyperproteic diet; NLChHFD, non-ketogenic low-carbohydrate high-fat diet; VLCRC, very-low-carbohydrate restricted calorie; LCMD, low-carbohydrate Mediterranean-style diet; LCM, low-carbohydrate Mediterranean; LChD, low-carbohydrate diet; LCHF, low-carbohydrate, high fat; LCHO diet, low-carbohydrate diet; LowCHO diet, low-carbohydrate diet; VLCD, very-low-carbohydrate diet; VLChD, very-low-carbohydrate diet; VLChHPD, very-low-carbohydrate hyperproteic diet; Very LCHF, very-low-carbohydrate/high-fat; KD, ketogenic diet; LCK, low-carbohydrate, ketogenic; LCKD, low-carbohydrate, ketogenic diet; VLCK, very-low-carbohydrate ketogenic; and VLCKD, very-low-carbohydrate ketogenic diet.

The wide variability in CRD proposals has led to the creation of a diversity of denominations, acronyms, and terms (e.g., very-low-, low-, moderate-, and high-carbohydrate diets; very poor, poor, moderate, or high in carbohydrates), as illustrated in Figure 2.

The concept and message conveyed by Figure 2 highlight the significant variability and lack of standardization in the terminology used to describe carbohydrate-restricted diets (CRDs). This diversity of acronyms and terms demonstrates the complexity and potential confusion surrounding the classification of CRDs and emphasizes the need for a more consistent and standardized approach to naming and categorizing CRDs, in order to ensure clearer communication and better understanding within the scientific community and beyond [13,45,46,47,48,49,50,51,52,53,54,55,56,57,58,59,60,61,62,63,64,65,66,67,68,69,70,71,72,73,74,75,76,77,78,79,80,81,82].

It is argued that this variety of nomenclatures and, consequently, acronyms, designating CRDs can generate ambiguity due to a lack of standardization, ultimately hindering understanding. This confounding factor may be related to the increase in the proportions of a particular macronutrient in the diet or the creation of denominations that combine carbohydrate restriction with specific dietary patterns, such as the low-carbohydrate Mediterranean diet.

Table 2 shows the most popular CRDs, their recommended carbohydrate contents, general characteristics, and food composition.

As shown in Table 2, some popular diets provide detailed guidance on which foods to consume and avoid, which is essential to promote adherence, given that people eat foods rather than nutrient percentages. For example, patterns like Atkins, which restrict all types of fruits, vegetables, milk, etc., may have implications in terms of lack of fiber and vitamins, as well as difficulty in adherence.

### 3.3. CRDs in Recommendations for Individuals with T2DM

CRDs are discussed in T2DM guidelines from several countries. International guidelines mention challenges in interpreting study results, owing to the broad range of definitions for elaborating carbohydrate-restricted food plans, which makes it difficult to generalize the results [1,5,6,85]. Classifications, carbohydrate percentages, and minimum and maximum carbohydrate contents of CRDs found in guidelines by Ramos et al. [8], Diabetes UK [86], Diabetes Canada [87], Diabetes Australia [88], and ADA [89]; the consensus of associations by Davies et al., [90]; and expert consensus by Evert et al. [4] are compiled in Table 3 and described in detail in Table S2 (Supplementary Material).

In 2011 and 2018, Diabetes UK issued statements concluding there was insufficient scientific evidence to promote a specific dietary approach or establish the ideal proportion of energy intake from fats, proteins, and carbohydrates. The organization argued that adherence is the best indicator of the long-term success of a dietary plan, underscoring the importance of individualizing approaches. However, it mentioned carbohydrate restriction as an appropriate option within this context. In 2017, Diabetes UK supported the adoption of what they called low-carbohydrate diets, although with caveats as to the uncertainty about the long-term effect of these approaches [6,91,92].

In 2018, a joint consensus was published between the ADA and EASD. This document cited low-carbohydrate diets for the first time as a therapy for glycemic control, with the advantage of having no side effects. Moreover, the organizations defined studies about low-carbohydrate diets as those addressing diets with <26% of total energy from carbohydrates [3]. The authors of the three systematic review and meta-analysis studies analyzed by Evert et al. [4] conclude that CRDs may have significant results for A1C for up to six months. It is noteworthy that this result comes from comparative analyses between low carb, high carb, and low fat diets, emphasizing that the effects tend to cease after one year for any of the tested diets, likely due to low adherence. The following year, another ADA communication highlighted that reducing total carbohydrate intake was the strategy with the strongest evidence for improving blood glucose levels in individuals with T2DM. In the consensus, they acknowledged the absence of a consistent definition for “low-carbohydrate” diets but categorized diets with a low carbohydrate content as one where 26% to 45% of total energy comes from carbohydrates. A very-low-carbohydrate diet was defined as one where carbohydrates are limited to <26% of total energy intake, with a goal of 20–50 g of carbohydrates per day to induce nutritional ketosis [4]. In the last consensus between the ADA and EASD on the management of hyperglycemia in T2DM, published in 2022 [90], it was cited that a Mediterranean or low-carbohydrate diet (<26% of total energy intake) provided the greatest benefits for glycemic control up to 6 months. This statement adopted the same classification as in 2018 [3] but diverged from the ADA’s own recommendation in 2019 [4].

Statements from international societies regarding the use of low-carbohydrate diets for individuals with diabetes emphasized the lack of a standardized definition in the literature for the carbohydrate content of such diets [86,87,88]. The position of the Australian guideline (2018) is based on two systematic reviews and meta-analyses [11,14], which used the definitions proposed by Feinman et al. [24]. This organization stated that low-carbohydrate diets are more effective in reducing average blood glucose levels in the short term. However, they underscored that the lack of definition for a low-carbohydrate diet makes it difficult to compare studies [88]. Diabetes UK (2021) [86] and SBD 2022/2023 (Ramos et al., 2022) [8] documents adopted the same definitions.

Diabetes Canada (2020) stated that individuals with T2DM should be encouraged to choose healthy eating patterns, including low- or very-low-carbohydrate diets. It was argued that such diets are effective for weight loss, glycemic control, and reducing the need for antihyperglycemic therapies [87]. However, it should be noted that the <45% carbohydrate/day recommendation can generate confusion, given that, depending on the total energy value of the diet, the carbohydrate intake can exceed 130 g (for example, 130 g of carbohydrates corresponds to 43% of the energy value of a 1200 kcal diet). That is, any diet with >1200 kcal would no longer be classified as low in carbohydrates. Furthermore, such definitions differ from those adopted by Feinman et al. [24], who classified diets with 26 to 45% carbohydrates as having moderate carbohydrate content, not low, which can lead to confusion when classifying and comparing studies.

The latest report from the British Scientific Advisory Committee on Nutrition (SACN), published in 2021 with Diabetes UK, revealed there is scientific evidence that CRDs (referred to as low-carbohydrate diet, with intakes of 50 to 130 g/day) are effective in improving glycemic control, serum triglyceride concentrations, and weight loss in people with T2DM. However, the report concluded that it was not possible to evaluate the impact of a diet called “low” compared to a diet called “high” in carbohydrates, on markers and outcomes of T2DM, given the wide variation in CRD definitions in randomized clinical trials [86]. SACN mentioned that the prescribed carbohydrate intake ranges from 14 to 50% of total energy. There was also an overlap in the means of carbohydrate intake between the “low-” (13–47% of total energy) and “high-” (41–55% of total energy) carbohydrate diets. Therefore, the comparisons were mainly between diets with lower and higher carbohydrate contents, rather than low- and high-carbohydrate diets [86].

In 2022, the SBD (2022) presented different designations for the same dietary classification of CRD published in the 2019 official guideline [8]. The organization currently adopts the classification of Feinman et al. [24], as do other societies. In the referred guideline, the terms moderate restriction (low-carbohydrate diet), intense restriction (very-low-carbohydrate diet), and extreme restriction (very low ketogenic diet) were used [8]. The novelty of the terms, which are not found in any other official document, can enhance the difficulty in classifying and interpreting such diets.

### 3.4. Definitions of CRDs and Impact Assessment Studies in Individuals with T2DM

We analyzed 12 review studies [9,10,11,12,13,14,15,16,17,18,19,20] investigating the impact of CRDs on health outcomes in individuals with T2DM. It was common for studies to use terms such as “low-carbohydrate diet scores” and “low-carbohydrate diet patterns”, even when the carbohydrate intake exceeded the limits of a given reference. These terminologies can lead to misinterpretations, making it difficult to use studies to reach conclusions about the safety and efficacy of low-carbohydrate diets.

The use of diverse terms may be due to the variety of diet compositions, hindering result interpretation and comparison. Furthermore, not all studies provided detailed information about the control diets. Among studies reporting this information, it was observed that the macronutrient composition of control diets differed greatly. Nevertheless, most studies used the definitions and classifications of CRDs proposed by Feinman et al. [24]. Of note, the referred study did not address food composition, only the macronutrient content of the proposed diets. Halton et al. [35,36] developed a scoring system for classifying low-carbohydrate diets based on a food frequency questionnaire. Currently, this system is little used and only applied to assess the consumption and impact of macronutrients, not to generate dietary recommendations.

The 12 review studies underscored that the impact of increasing the proportions of other macronutrients (usually fats and/or proteins) to compensate for reduced carbohydrate intake is often not considered in scientific studies. Nor is the type of macronutrient (e.g., saturated or unsaturated fats) taken into account when evaluating markers and clinical outcomes of T2DM [12,13,14,16,20]. Three review studies reported that none of the evaluated studies provided information on the type of carbohydrate consumed (for example, whole grains, refined grains, free sugars, and fiber) or considered how this factor could affect the results of interest [14,17,20]. It is also worth noting that none of the review studies assessing the impact of CRD on health outcomes in individuals with diabetes mellitus reported food composition or investigated how this factor may influence diet adherence and health outcomes [9,10,11,12,13,14,15,16,17,18,19,20].

### 3.5. Food Composition of CRDs for Individuals with T2DM

According to the ADA, the acceptable dietary patterns for diabetes control, including CRDs, should be based on the following recommendations: (i) emphasize the consumption of non-starchy vegetables, (ii) minimize the consumption of added sugars and refined grains, and (iii) choose whole-grain foods over highly processed foods [4]. It should be noted, however, that even though simple carbohydrates have the greatest glycemic impact, especially added sugars, the ADA suggests minimizing, rather than avoiding, their consumption. Furthermore, the opposition of whole foods to highly processed foods is questionable, given that a food can be deemed whole grain and at the same time be highly processed. The recommendation could be to choose fresh or minimally processed foods, preferably whole grain, rather than highly processed foods.

Regarding the 12 review studies [9,10,11,12,13,14,15,16,17,18,19,20], the lack of information about food composition may compromise the results. Given that the aim of such studies was to analyze the effect of a certain diet, it would be essential that information about diet definitions, macronutrient contents, and food composition be clearly stated. It was observed that the denomination “low-carbohydrate diet” is widely used, regardless of diet characteristics. Churuangsuk et al. [93] agreed that this approach may lead to variations in foods and metabolic responses, which may differ according to the food source of the macronutrient.

Westman et al. [44] argued that the variability in dietary intervention methodologies may explain, but not justify, the lack of information on the food composition of diets. Some studies used a diet book, dietitian’s recommendations, or leaflets to instruct participants about the quantities and types of foods [44]. Other studies used a list of permitted foods [94] and/or an ad libitum consumption strategy. Participants were provided with information on menu planning, sample menus, guidelines for eating out, and food preparation, an interactive planner, and cooking demonstrations, in addition to other educational materials. Such approaches were adopted with the goal of increasing engagement, supporting behavior change, and ensuring diet adherence [95]. However, in these cited cases, food evaluation was conducted only to determine whether carbohydrate restriction had been followed.

Researchers have the flexibility to select the most suitable method for their studies. However, an analysis of a diet’s impact on health must include detailed information about the foods comprising these diets; otherwise, the validity of the results may be compromised. Discussions on this topic are limited, and even the quality assessment tools for review studies do not appear to adequately address its significance, as these tools are not tailored to food and nutrition.

Noakes and Windt [94] explained that CRDs (referred to by the authors as low-carbohydrate, high-fat diets) are typically defined by what is excluded rather than what is included. Studies often emphasize the restrictions but may not adequately account for or control the types of foods that are actually consumed. Although the details may vary depending on the specific type of CRD (e.g., Atkins, Banting, Paleo, or South Beach), in each of these diets there is a consistent focus on unprocessed foods, such as cruciferous vegetables, leafy greens, raw nuts and seeds, eggs, fish, unprocessed animal meats, dairy products, and natural vegetable oils and fats from avocados, coconuts, and olives.

The mentioned authors argued that low-carbohydrate, high-fat diets do not lack carbohydrates, even ketogenic diets. On the contrary, the consumption of leafy greens, cruciferous vegetables, and other non-starchy vegetables is encouraged, along with a moderate intake of berries. Table 4 shows a list of foods of the Banting diet (low-carbohydrate, high-fat) proposed by Noakes et al. [96]. It is observed that the diet focuses on vegetables and animal proteins. The dietary plans of low-carbohydrate, high-fat diets include meals such as omelets, salads, and animal proteins such as steak, salmon, or chicken served with vegetables.

Current evidence indicates that most studies employing carbohydrate-restricted dietary patterns did not limit saturated fat intake, and this approach does not appear to increase overall cardiovascular risk [4]. Thus, further investigation into the nutritional composition of these diets could provide a comprehensive understanding of their health impacts while addressing concerns about dietary fat intake [48,60,66,68,70,72,78,80,81].

Most very-low-carbohydrate ketogenic diets are based on completely avoiding all starchy carbohydrates and sugars, and consuming low-carbohydrate fruits, vegetables, and leafy greens. Such diets promote an increased consumption of fats and proteins, such as meat, fish, eggs, and cheese. Dairy products with higher contents of carbohydrates, for instance, milk and yogurt, are generally avoided, although most cheeses and butter are included because they contain minimal or no carbohydrates [17].

Among the official diabetes guidelines and documents, only Diabetes Australia provides practical food considerations for individuals with diabetes (type 1 diabetes mellitus and T2DM) adopting CRDs. One of these considerations is to restrict the consumption of high-energy foods and drinks that are high in carbohydrates and low in nutrients, such as sugary drinks, snacks, cakes, cookies, sweets, and confectionery. Furthermore, it is suggested to include foods known to be beneficial to health, such as vegetables, fruits, whole grains, dairy products, nuts, legumes, seafood, meat, and eggs [85]. Diabetes Australia seems to restrict only highly processed foods and does not address other foods, for example, starches, vegetables, and fruits with different carbohydrate contents or dairy products with different fat and carbohydrate contents.

In general, recommendations are to regularly review and customize nutritional orientations for individuals with T2DM. The ADA developed guidance material for healthcare professionals regarding the food composition of CRDs for individuals with T2DM [97]. This was the only document of its kind found in the literature; however, it was not included in the current study due to its paid access, which may greatly limit its availability to healthcare professionals.

### 3.6. Evaluation of the Positive and Negative Aspects of CRDs for Individuals with T2DM

**Figure 3:**
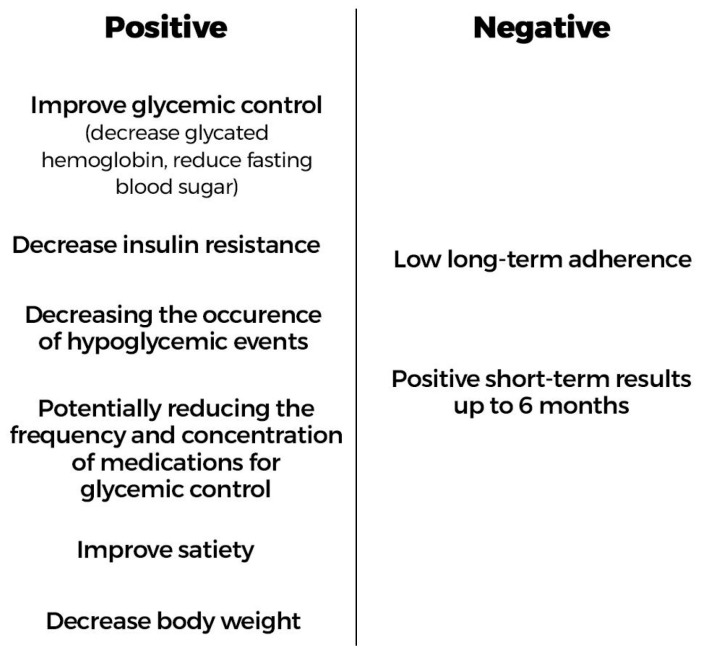
Positive and negative aspects of CRDs for individuals with T2DM. Source: [23,24,25,26,27].

Based on the findings of this scoping review, the positive and negative aspects of CRDs for individuals with T2DM are summarized in Figure 3.

The positive aspects include the results in controlling the disease from a metabolic point of view. The negative aspects are interconnected, as studies show that the diet has low long-term adherence, which is probably the reason why the results of CRDs are only maintained for up to 6 months.

Precisely, interventions using low-carbohydrate dietary plans often face challenges with regard to long-term adherence. On the one hand, it is considered that the limitations already discussed in the studies make it difficult to carry out an in-depth analysis of the supposed difficulties in adhering to CRDs. On the other hand, studies analyzing the narratives of experiences of individuals with T2DM with CRDs mainly find a lack of reliable information about CRDs and a lack of public policies, health professionals, and community and family support to follow the diet. Individuals with T2DM report feeling alone in following the CRDs [27,80,98,99]. A Canadian study showed that dietitians feel a lack of training to prescribe CRDs and to support individuals with T2DM [100].

### 3.7. Limitations

This scoping review has some limitations inherent to the method. For example, only articles published in English, Portuguese, or Spanish were included. This linguistic restriction may influence the overall understanding of the results and introduce a bias due to the possible exclusion of valuable contributions published in other languages referring to other cultures. Furthermore, it was often difficult to access the original versions of official documents. There were some challenges to understanding and organizing the great variety of terms for CRDs. Finally, there was little information about the food composition of CRDs.

### 3.8. Implications and Recommendations for Future Studies

It is essential to standardize CRD definitions due to the current lack of consensus. Establishing uniform definitions and categorizations for varying levels of carbohydrate restriction should be prioritized to enhance clarity and consistency across studies. Such an approach would allow the comparison of results, ultimately supporting evidence-based dietary recommendations. Furthermore, research on CRDs must provide detailed reporting of food composition, including not only macronutrient distribution but also specific food sources. This approach acknowledges that people consume foods, not macronutrient percentages, and that dietary habits are deeply influenced by local cultures. Addressing these methodological considerations during study design can provide a more comprehensive understanding of how different foods affect glycemic and metabolic responses. Such improvements could significantly deepen insights into the relationship between dietary components and health outcomes.

In view of the above, it is proposed that such diets be generically referred to as CRDs. In this dietary approach, it is assumed that the high satiety provided by high protein and fat intake naturally leads to a quantitative restriction of carbohydrates. Furthermore, it is necessary to provide information on which carbohydrate types and sources should be avoided, considering their glycemic index. For instance, simple sugars, starches, fruits with a high glycemic index, and vegetables with more than 20% carbohydrates (e.g., carrots, pumpkin, beets, and potatoes) should be limited in daily meals, in addition to highly processed foods.

Individualized approaches, food-based dietary guidelines, and evidence-based recommendations for healthcare professionals are needed for more effective integration between research and clinical practice in guiding patients interested in CRDs.

## 4. Conclusions

The analysis of CRD definitions and classifications revealed a lack of consensus and standardization, characterized by an excess of designations (e.g., usual diet, high-carbohydrate diet, moderate-carbohydrate diet, low-carbohydrate diet, very-low-carbohydrate diet, very-low-carbohydrate ketogenic diet) and, consequently, acronyms, which can limit the understanding and use of these dietary approaches. The absence of scientifically accepted criteria for categorizing these diets poses challenges in the interpretation and comparison of study results. In evaluating the review studies about the impact of CRDs on the health outcomes of individuals with T2DM, it was observed that the most common designation for CRDs was low-carbohydrate diet, characterized as <26% or 130 g/day, as proposed by Feinman et al. [24]. Nevertheless, the cited study underscored the need for a more uniform terminology.

The diets analyzed here were defined according to the amount of carbohydrates relative to total energy value, ranging from <26% to <45%. With this approach, the actual carbohydrate intake may vary according to the total energy value of the diet. For the same reason, defining a CRD based solely on the grams of carbohydrates per day does not guarantee carbohydrate restriction for different profiles of individuals. Thus, it becomes necessary to analyze the food composition of the diet. Conversely, the foods consumed by individuals were the least discussed aspect in all studies evaluated here. Dietary recommendations from the scientific literature and official documents have the same limitation.

Among our objectives was to analyze the differences between the tested diets and the results considering the food composition. However, it was not possible to have this discussion because the studies do not provide this information, and perhaps this is one of the reasons why the long-term difference analyses between the diets are inconclusive.

The divergence in definitions, coupled with the lack of detailed information on food composition in interventions, highlights the need for more specific and methodologically consistent studies. Diets based on fresh or minimally processed foods should be analyzed for inclusion in the decision making and recommendations of official diabetes guidelines and organizations. The nuances raised here may support future studies adopting homogeneous classifications and methodological designs. Such efforts may provide more consistent data on CRDs for decision making and practical recommendations by official documents and bodies for the treatment of T2DM. This information may also support the development of public policies, potentially improving the management and performance of professionals caring for individuals with T2DM.

## Figures and Tables

**Table 1:** Classification and parameters of carbohydrate-restricted diets considering different carbohydrate contents.

Class	Carbohydrate Intake		Protein Intake	Fat Intake
	g/day	% Total Energy ^1^	% Total Energy ^1^	% Total Energy ^1^
Very-low-carbohydrate diet [considered a ketogenic diet]	21 to 70 ^2^ 20 to 50 ^4^	4 to 14 ^2^ 0 to 20 ^3^ ≤10 ^4^	-	-
Very-low-carbohydrate hyperproteic diet	-	0 to 20 ^3^	55 to 65 ^3^	25 to 35 ^3^
Low-carbohydrate diet	150 to 200 ^1^ >50 to <130 ^4^	30 to 39.99 ^2^ 20 to 40 ^3^ >10 to ≤26 ^4^	-	-
Non-ketogenic low-carbohydrate diet	-	20 to 40 ^3^	20 to 30 ^3^	30 to 60 ^3^
Low-carbohydrate hyperproteic diet	-	20 to 40 ^3^	30 to 60 ^3^	20 to 30 ^3^
Moderate-carbohydrate diet	200 to 325 ^2^ 130 to 230 ^3^	40 to 65 ^2^ 26 to 45 ^3^	-	-
High-carbohydrate diet	>325 ^2^ >230 ^4^	>65% ^2^ >45 ^4^	-	-

^1^ Based on a 2000 kcal/day diet; ^2^ Kirk et al. (2008) [34]; ^3^ Frigolet, Ramos Barragán, and Tames González (2011) [39]; ^4^ Adapted by Feinman et al. (2015) [24] from Accurso et al. (2008) [23] and the references contained therein.

**Table 2:** Description of popular carbohydrate-restricted diets for DM2 *.

Diet	Carbohydrate Content **	Macronutrient Composition (Total Energy Basis)	Characteristics	Foods Allowed	Foods to Avoid
Atkins	Induction phase: 20 g/day; later phases: 80–100 g/day	Carbohydrate: 5% Protein: 27% Total fat: 68% Saturated fat: 26% Alcohol: 0%	Four phases with progressively lower restrictions. The “New Atkins” diet includes a 40 g/day induction phase option for those with <40 lb (18.14 kg) overweight.	Meat, fish, and poultry Eggs Cheese Low-carbohydrate vegetables Butter and oil	Breads and pastaMost fruits and vegetables Milk Alcoholic beverages
Ketogenic	<50 g/day	-	Patients can check their urine for ketones or request blood tests to confirm ketotic states (elevated β-hydroxybutyrate). The diet emphasizes a period of “keto-adaptation”, whereby the body switches from glucose to fat as the main source of energy.	-	-
Dr Bernstein’s Diabetes Solution	30 g/day	-	One of the original diets focused on the glycemic index, restricting foods that cause a rapid increase in blood sugar.	-	-
Eco-Atkins	130 g/day	-	Vegan diet containing 31% protein, 43% fat, and 26% carbohydrate.	-	-
Low carbohydrate, high fat (LCHF)	<20–100 g/day	-	Focused on fat intake to promote satiety.	-	-
Paleo	Varies with food choices	-	Limited to foods eaten by early humans.	Meat, fish, and eggs Vegetables, fruits, and nuts	Minimize whole grains Processed foods Foods with added sugars Dairy Legumes and potatoes
Protein Power	28–40 g/day	Carbohydrate: 16% Protein: 26% Total fat: 54% Saturated fat: 18% Alcohol: 4%	Focused on adequate protein intake and limited carbohydrate intake divided into 4 or 5 meals/snacks a day.	Meat, fish, and poultry Eggs Cheese Low-carbohydrate vegetables Butter, oil, and salad dressings Alcoholic beverages in moderation	Breads and pasta Fruits and vegetables Fats and oils Dairy products
South Beach	Phase 1: excludes most carbohydrates Phases 2 and 3: ≤140 g/day	-	Created in response to concerns about the high contents of saturated fat in the Atkins diet. Focused on restricting carbohydrates and saturated fats. Comprises three meals and three snacks a day.	-	-
Sugar Busters	2–3 servings a day	-	Focused on controlling the glycemic index by minimizing the intake of refined sugars, white flour, and starches.	-	-
Sonoma	Varies with food choices	-	Three phases focused on controlling serving sizes. Combines Mediterranean and low-carbohydrate diets. Minimizes the intake of saturated fat, starches, and sugar.	-	-
Stillman	-	Carbohydrate: 3% Protein: 64% Total fat: 33% Saturated fat: 13% Alcohol: 0%	-	Lean meats and skinless poultry meat Lean fish and seafood Eggs Skim milk, cottage cheese, and other cheeses	Breads and pasta Fruits and vegetables Fats and oils Dairy products Alcoholic beverages
Zone	40%	Carbohydrate: 36% Protein: 34% Total fat: 29% Saturated fat: 9% Alcohol: 1%	Focused on adequate proportions of carbohydrates, proteins (30%), and fats (30%) to aid in satiety and metabolism. The diet advocates small, frequent meals and snacks, totaling seven daily eating occasions. Protein, fat, and carbohydrates in exact proportions.	Foods with a low glycemic index Alcoholic beverages in moderation	Breads and pasta Some fruits Saturated fats

* Informations by Chandler [83] and Fields [84]. ** Reproduces exactly the information contained in the cited bibliographies.

**Table 3:** Diet denominations based on carbohydrate content, according to the guidelines of different countries for individuals with diabetes mellitus 2.

Classification	Carbohydrate % (Country)	Carbohydrate Content (Country)
Usual diet	45–65% (BRA ^2^)	Individualized (BRA ^2^)
High-carbohydrate diet	>45% (AUS ^6^, UK ^3^)	>230 g (UK ^3^)
Moderate-carbohydrate diet	26–45% (AUS ^6^, UK ^3^)	>225 g (AUS ^6^)
Low-carbohydrate diet	26–45% (BRA ^2^, USA ^5^) <45% (CAN ^4^)	130–230 g (UK ^3^) 50–130 g (CAN ^4^) <130 g (EUA ^7^)
Very-low-carbohydrate diet		130–225 g (AUS ^6^) <50 g (CAN ^4^)
Very-low-carbohydrate ketogenic diet	<26% (AU ^6^, UK ^3^, USA ^1^)	

Legend: AUS, Australia; BRA, Brazil; CAN, Canada; UK, United Kingdom; USA, United States of America. Source: ^1^ Davies et al. (2022) [90]; ^2^ Ramos et al. (2022) [8]; ^3^ Diabetes UK (2021) [86]; ^4^ Diabetes Canada (2020) [87]; ^5^ Evert et al. (2019) [4]; ^6^ Diabetes Australia (2018) [88]; ^7^ ADA (2007) [89].

**Table 4:** Recommended foods in the carbohydrate-restricted Banting diet (low-carbohydrate, high-fat) *.

Animal Protein	Dairy	Fats	Nuts and Seeds	Vegetables
Eggs	Cottage cheese	Olive oil	Almonds	All leafy greens
Meats	Cream	Avocado	Flaxseed	Cruciferous vegetables
Poultry	Whole milk cream	Coconut oil	Macadamia	Aboveground vegetables
Game	Whole milk Greek yogurt	Macadamia oil	Walnuts	
Seafood	Cheeses		Pine nuts	

* Information by Noakes et al. (2013) [96].

## Supplementary Materials

The following supporting information can be downloaded at https://www.mdpi.com/article/10.3390/nu17061061/s1: Table S1: Authors, year of publication, country, and main findings of the documents analyzed in this study, in ascending chronological order. Table S2: Classification and carbohydrate percentages of carbohydrate-restricted diets recommended for individuals with diabetes by guidelines of different countries.
