# Supplementary material for: Structural Concepts, Definition, Classification, and Macronutrient and Food Composition of Carbohydrate-Restricted Diets for Individuals with Type 2 Diabetes Mellitus: A Scoping Review
Source: Nutrients. 2025 Mar 18;17(6):1061. doi: 10.3390/nu17061061 (PMC11944602; doi:10.3390/nu17061061)
Supplement: Supplementary file 1 [file nutrients-17-01061-s001.zip › nutrients-3514024-supplementary.pdf]

**Table S1.** Authors, year of publication, country, and main findings of the documents analyzed in this study, in ascending chronological order.

| *N* | Authors, year | Country | Main findings | | |
|---|---|---|---|---|---|
| | | | Concepts or diet classification | Macronutrient composition of diets | Diet composition |
| 1 | Reaven GM, 1986 | USA | | x | |
| 2 | Parillo et al., 1992 | Italy | | x | x |
| 3 | Garg et al., 1994 | USA | | x | |
| 4 | Eades MR, Eades MD, 1996 | USA | | x | |
| 5 | Atkins RC, 2002 | USA | | x | x |
| 6 | Vernon MC, Eberstein JA, 2004 | USA | | x | |
| 7 | Baldwin EJ, 2004 | UK | | x | |
| 8 | Sheard et al., 2004 | USA | | x | |
| 9 | IOM, 2005 | Switzerland | | x | |
| 10 | Yancy et al., 2005 | USA | | x | |
| 11 | Halton et al., 2006 | USA | | x | |
| 12 | Last AR, Wilson SA, 2006 | USA | x | x | |
| 13 | Nielsen JV; Joensson E, 2006 | Sweden | | x | |
| 14 | Chandler MJ, Hildebrandt LA, 2007 | USA | | | x |
| 15 | ADA, 2007 | USA | | x | |
| 16 | Worth J, Soran H, 2007 | UK | | x | |
| 17 | Halton et al., 2008 | USA | | x | |
| 18 | Accurso et al., 2008 | USA | x | x | |
| 19 | Kirk et al., 2008 | USA | | x | |
| 20 | Volek et al., 2008 | USA | x | | |
| 21 | Westman et al., 2008 | USA | | x | |
| 22 | Wylie-Rosett J, Davis NJ, 2009 | USA | x | | x |
| 23 | Feinman RD, 2011 | USA | x | x | |
| 24 | Frigolet ME, Ramos Barragán VE, Tamez González M, 2011 | Mexico | | x | |
| 25 | Dyson et al., 2011 | UK | | x | |
| 26 | Hite et al., 2011 | USA | | x | |
| 27 | Hussain et al., 2012 | Kuwait | x | x | x |
| 28 | He et al., 2012 | China | | x | |
| 29 | Wheeler et al., 2012 | USA | x | | |
| 30 | Ajala O, English P, Pinkney J 2013 | USA | | x | |
| 31 | Noakes et al., 2013 | South Africa | | x | |
| 32 | Fernemark et al., 2013 | Sweden | | x | |
| 33 | Paoli et al., 2013 | USA | | x | |
| 34 | Haimoto et al., 2014 | Japan | | x | |
| 35 | Saslow et al., 2014 | USA | | x | |
| 36 | Czyzewsa-Majchrzak L, 2014 | Poland | | x | |
| 37 | Guldbrand et al., 2014 | Sweden | | x | |
| 38 | Dyson P, 2014 | UK | | x | x |

| | | | | | |
|---|---|---|---|---|---|
| 39 | Feinman et al., 2015 | USA | x | x | |
| 40 | Hernandez et al., 2015 | Mexico | | x | |
| 41 | Dyson P, 2015 | UK | | x | |
| 42 | Van WYK, Davies RE, Davies JS, 2015 | UK | | x | |
| 43 | RACGP, 2016 | Australia | | x | |
| 44 | Fields et al., 2016 | USA | | x | x |
| 45 | Mc Ardle PD et al., 2016 | UK | | x | |
| 46 | Sato J et al., 2016 | Japan | | x | |
| 47 | Diabetes UK, 2017 | UK | | x | |
| 48 | Meng et al., 2017 | China | | x | |
| 49 | Snorgaard et al., 2017 | Denmark | | x | |
| 50 | Noakes TD, Windt J, 2017 | Canada | | x | x |
| 51 | Yamada S, 2017 | Japan | x | x | |
| 52 | Dyson et al., 2018 | UK | | x | |
| 53 | Davies et al., 2018 | USA, UK | | x | |
| 54 | Huntriss R, Campbell M, Bedwell C, 2018 | UK | | x | |
| 55 | Liu K et al., 2018 | China | | x | x |
| 56 | Sainsbury et al., 2018 | Australia | | x | x |
| 57 | Sievenpiper et al., 2018 | Canada | | x | |
| 58 | Van Zuuren et al., 2018 | Netherlands | x | | |
| 59 | Diabetes Australia, 2018 | Australia | | x | |
| 60 | Tay J et al., 2018 | Australia | | x | |
| 61 | Shafique M et al., 2018 | UK | | x | x |
| 62 | Evert et al., 2019 | USA | | x | |
| 63 | Huntriss R, Boocock R, McArdle P, 2019 | UK | | x | |
| 64 | Korsmo-Haugen et al., 2019 | Norway | | x | |
| 65 | Kirkpatrick CF et al. 2019 | USA | | x | |
| 66 | McArdle et al., 2019 | UK | | x | |
| 67 | SBD, 2020 | Brazil | | x | |
| 68 | Dyson P, 2020 | UK | | x | |
| 69 | Diabetes Canada, 2020 | Canada | | x | |
| 70 | Tay J et al., 2020 | Australia | | x | x |
| 71 | Churuangsuk C, Lean M, Combet E, 2020 | UK | | x | |
| 72 | Kelly T, Unwin D, Finucane F, 2020 | Ireland | | x | |
| 73 | Westman EC, Yancy JR, 2020 | USA | x | x | x |
| 74 | Foley PJT, Gunson JTS, Baumann SL, 2020 | UK | | x | x |
| 75 | Chen CY et al., 2020 | Taiwan | | x | |
| 76 | Merril JD et al., 2020 | USA | | x | |
| 77 | Wheatley et al., 2021 | UK | x | x | |
| 78 | Singh et al., 2021 | UK | | x | |
| 79 | Wong et al., 2021 | Canada | | x | x |
| 80 | Dashti HM, Mathew TC, Ai-Zaid NS, 2021 | Kuwait | | x | x |
| 81 | Diabetes UK, 2021 | UK | | x | |
| 82 | Skytee MJ et al., 2021 | Denmark | | x | |

| | | | | |
|---|---|---|---|---|
| 83 | Wong K et al., 2021 | Canada | x | |
| 84 | Davies et al., 2022 | USA, UK | x | |
| 85 | Dening et al., 2022 | Australia | x | x |
| 86 | Goldnberg JZ et al., 2022 | USA | x | |
| 87 | Jayedi et al., 2022 | Iran | x | |
| 88 | Neudorf H et al., 2022 | Canada | x | x |
| 89 | Ramos et al., 2022 | Brazil | x | |
| 90 | Sun et al., 2022 | Global | x | |
| 91 | Siverhus K, 2022 | USA | x | |
| 92 | Scoot E et al., 2022 | UK | x | |
| 93 | Kumar NK et al., 2022 | USA | x | x |
| 94 | Li S et al., 2022 | China | x | x |
| 95 | Gram-Kampmann EM et al., 2022 | Denmark | x | |
| 96 | ADA, 2024 | USA | x | |

Legend: Official documents; ADA, American Diabetes Association (ADA); USA, United States of America; UK, United Kingdom; SBD, Brazilian Diabetes Society.

**Table S2.** Classification and carbohydrate percentages of carbohydrate-restricted diets recommended for individuals with diabetes by guidelines of different countries.

| Country | Organization, document | Classification | Carbohydrate content relative to total energy value (%) | Total carbohydrate intake (g/day) |
|---|---|---|---|---|
| Australia | Diabetes Australia (2018)[6]: Position statement on low-carbohydrate diets for individuals with diabetes mellitus | High-carbohydrate diet | <45% | >225 |
| | | Moderate-carbohydrate diet | 26%–45% | 130–225 |
| | | Low-carbohydrate diet | <26% | <130 |
| Brazil | Brazilian Diabetes Society (2023)[2]: Guidelines | Usual diet | 45%–65% | Individualized |
| | | Moderate restriction, low-carbohydrate diet | 26%–45% | <130 |
| | | Intense restriction, very-low-carbohydrate diet | <26% | Individualized |
| | | Extreme restriction, very-low-carbohydrate ketogenic diet | <10% | 20–50 |
| Canada | Diabetes Canada (2020)[4]: Position statement on low-carbohydrate diets for adults with diabetes: A quick review | Low-carbohydrate diet | <45% | 50–130 |
| | | Very-low-carbohydrate diet | - | <50 |
| USA | American Diabetes Association[7] | Low-carbohydrate diet | <26%[1] | <130[7] |
| | | | 26%–45%[5] | - |
| | | Very-low-carbohydrate diet | <26%[5] | 20–50[5] |
| United Kingdom | Diabetes UK (2021)[3]: | High-carbohydrate diet | >45% | >230 |

| | | | |
|---|---|---|---|
| Diets with low carbohydrate content for adults with T2DM | Moderate-carbohydrate diet | 26%–45% | 130−230 |
| | Low-carbohydrate diet | <26% | <130 |
| | Very-low-carbohydrate ketogenic diet | ≤10% | 20–50 |

Source: [1]Davies et al. (2022), [2]Ramos (2022); [3]Diabetes UK (2021); [4]Diabetes Canada (2020); [5]Evert et al. (2019), [6]Diabetes Australia (2018), [7]ADA (2007).
